# Optimizing methadone dose adjustment in patients with opioid use disorder

**DOI:** 10.3389/fpsyt.2023.1258029

**Published:** 2024-01-08

**Authors:** Po-Shen Liu, Teng-Yao Kuo, I-Chun Chen, Shu-Wua Lee, Ting-Gang Chang, Hou-Liang Chen, Jun-Peng Chen

**Affiliations:** ^1^Department of Psychiatry, Taichung Veterans General Hospital, Taichung, Taiwan; ^2^Fundamental General Education Center, National Chinyi University of Technology, Taiping, Taiwan; ^3^Faculty of Medicine, National Yang Ming Chiao Tung University, Taipei, Taiwan; ^4^Department of Post-Baccalaureate Medicine, College of Medicine, National Chung Hsing University, Taichung, Taiwan; ^5^Tsaotun Psychiatric Center, Ministry of Health and Welfare, Nantou, Taiwan; ^6^Biostatistics Task Force of Taichung Veterans General Hospital, Taichung, Taiwan

**Keywords:** methadone, opioid use disorder, telemedicine, dose optimization, mixed model

## Abstract

**Introduction:**

Opioid use disorder is a cause for concern globally. This study aimed to optimize methadone dose adjustments using mixed modeling and machine learning.

**Methods:**

This retrospective study was conducted at Taichung Veterans General Hospital between January 1, 2019, and December 31, 2020. Overall, 40,530 daily dosing records and 1,508 urine opiate test results were collected from 96 patients with opioid use disorder. A two-stage approach was used to create a model of the optimized methadone dose. In Stage 1, mixed modeling was performed to analyze the association between methadone dose, age, sex, treatment duration, HIV positivity, referral source, urine opiate level, last methadone dose taken, treatment adherence, and likelihood of treatment discontinuation. In Stage 2, machine learning was performed to build a model for optimized methadone dose.

**Results:**

Likelihood of discontinuation was associated with reduced methadone doses (*β* = 0.002, 95% CI = 0.000–0.081). Correlation analysis between the methadone dose determined by physicians and the optimized methadone dose showed a mean correlation coefficient of 0.995 ± 0.003, indicating that the difference between the methadone dose determined by physicians and that determined by the model was within the allowable range (*p* < 0.001).

**Conclusion:**

We developed a model for methadone dose adjustment in patients with opioid use disorders. By integrating urine opiate levels, treatment adherence, and likelihood of treatment discontinuation, the model could suggest automatic adjustment of the methadone dose, particularly when face-to-face encounters are impractical.

## 1 Introduction

In 2016, the global estimate indicated that 26.8 million individuals were living with opioid use disorder (1). Apart from essential harm reduction, patients dependent on opioids are usually encouraged to initiate into opioid agonist treatment (2), with therapeutic options such as medically supervised withdrawal or a multi-interventional approach in residential therapeutic community considered depending on severity and chronicity of opioid use disorder (3, 4). In Taiwan, methadone-based opioid agonist treatment entails daily visits to clinics for medication administration, overseen by a nurse. The advent of the COVID-19 pandemic in 2019 has compelled adjustments in methadone treatment approaches (5), such as incorporating mobile outreach (6), waiving urine toxicology screening, and authorizing take-home methadone (7–9). Adapting opioid agonist treatment to the evolving landscape during the pandemic is imperative, necessitating flexibility while upholding treatment effectiveness.

Telemedicine has advantages such as offering options for individuals whose life circumstances make in-person treatment difficult and whose work schedules make the logistics of in-person care challenging (10). Studies have validated telemedicine as a substance treatment services (7, 10). This inspired our efforts to launch virtual visits for the treatment of opioid use disorders. Physicians adjust the methadone dose depending on the patient’s tolerance and opioid withdrawal symptoms. Dose optimization based on artificial intelligence has been applied to patients with type 1 diabetes, and for opioid infusion during general anesthesia (11–13). We hypothesized that dose optimization for opioid agonist treatment involving methadone could be developed using clinical parameters.

The protocol of opioid agonist treatment involving methadone commences usually with a daily dose in the range of 10–30 mg methadone (14). At the induction phase, the dose is adjusted gradually by adding 5–10 mg every 3–5 days (15). The dose should be increased in an adjusted tempo allowing patients to keep avoid using illicit opioids, and avoid toxicity. After obtaining steady state, the dose adjusting can be made more gradually with intervals of 1–2 weeks. The most effective dose for retaining patients and reducing heroin use ranges from 60 to 100 mg/day (16).

For individuals who persist in using illicit opiates despite being prescribed within this dose range of methadone, administrating doses greater than 100 mg daily may be considered (17). Using a stable effective dose is of critical significance to keep patients retained in the treatment. For successful withdrawal from opioid agonist maintenance, the decision to reduce dosage and stop treatment should consider comprehensively, including a positive change of social condition and cessation of illicit drug use (18). The duration of opioid agonist treatment should not be limited to some months or years. Such standardization of the treatment duration and dose reduction suggestions may increase the risk of relapse and overdoes death.

Previous studies reported that methadone dose increase had beneficial therapeutic effects, including reduced frequency of opiate use and craving (19). A previous study showed that in patients receiving opioid agonist treatment, a diminishing dose trend led to the cessation of opioid agonist treatment (20). In other words, patients receiving sub-therapeutic doses are more likely to discontinue their treatment. The likelihood of treatment discontinuation can be evaluated by self-reporting or physicians (21). In cases where face-to-face encounters are impractical, the likelihood of treatment discontinuation is indicated by deviation from the treatment protocol. In this regard, if a patient does not want to increase the methadone dose when urine drug screening results are positive for opiates, the likelihood of treatment discontinuation may increase.

Here, we aimed to examine whether treatment adherence, urine opiate test results, and the likelihood of treatment discontinuation are valuable metrics for determining methadone dose. We also developed a model for optimizing the methadone dose adjustment, which may be applied to opioid agonist treatment.

## 2 Methods

### 2.1 Participants

**Figure 1:**
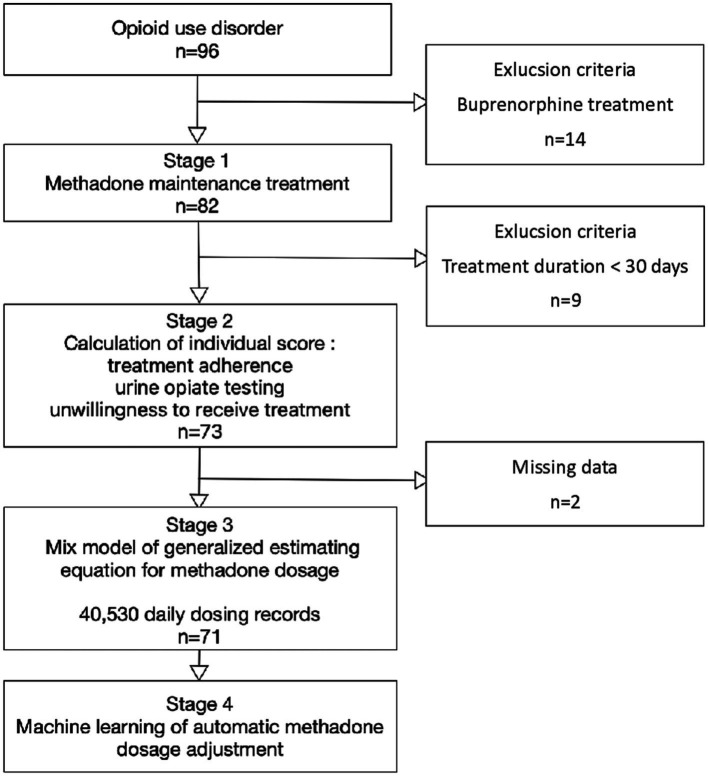
Flow chart of the study design.

This retrospective study of opioid agonist treatment was conducted at Taichung Veterans General Hospital between January 1, 2019, and December 31, 2020. The patients underwent weekly clinic visits during the initial treatment period. Monthly clinic visits were required for methadone dosing starting from the second month of treatment. We obtained time-series data on the individual daily dosages of 96 eligible subjects receiving opioid agonist treatment. The inclusion criterion was opioid use disorder according to the diagnostic criteria of the Diagnostic and Statistical Manual of Mental Disorders 5th Edition (DSM 5). The exclusion criteria were total treatment time < 30 days and receiving buprenorphine instead of methadone (Figure 1). For patients who discontinued treatment during the induction phase, it could be considered a lack of data during the maintenance treatment period, akin to the concept of missing data. The study protocol was approved by the Ethics Review Committee of the Taichung Veterans General Hospital (project number *CF*-19714A).

#### 2.1.1 Treatment protocol

At Taichung Veterans General Hospital, the standard procedure for opioid agonist treatment involving methadone typically initiated with a daily dosage ranging from 10 to 40 mg. The initial month of treatment constituted the induction phase, during which the methadone dosage underwent gradual adjustments by incorporating increments of 5–10 mg every 3–7 days. These adjustments persisted until the individual ceased experiencing withdrawal symptoms.

Upon entering the maintenance phase, the methadone dose was stabilized at a target range of 60–100 mg, Patients were encouraged to take higher doses of methadone in response to craving, withdrawal symptoms, or positive opiate testing. Moreover, patients have the autonomy to progressively reduce their methadone dosage by 2.5–5 mg each month upon exhibiting positive changes in their social and physical wellbeing, cessation of illicit drug use, and the attainment of stable employment.

### 2.2 Collected data

#### 2.2.1 Treatment adherence

The database for methadone dispensing included dosing records, daily methadone dosage, missed dosages, and leave applications. The total number of dosing days at the hospital during the past 30 days was automatically calculated. The total number of days attended during the past 30 days was represented by $\chi_{1}$ , which took on values from 0 to 30.

#### 2.2.2 Urine drug screen

The level of illicit opiate in urine drug screen for were conducted every 3 months. Qualitative testing was conducted using an enzyme multiplied immunoassay technique (EMIT). Cutoffs for positive specimens were 300 ng/mL for opiate. A previous study suggested measuring reductions in the extent of drug use using a score rather than a categorical assignment to achieve complete abstinence (19). Urine opiate results within the last 90 days were represented by $\chi_{2}$ , which was automatically coded as either 10 for opiate-positive or 30 for opiate-negative results. Each positive urine test would be counted as 10. Each negative urine test would be counted as 30. For patients whose urine drug screening results were positive for opiates, an add-on urine drug screen a week later was accomplished. If the add-on urine drug screening results were negative for opiates, $\chi_{2}$ was coded as 20. If the add-on urine drug screening results were positive for opiates, $\chi_{2}$ was coded as 10.

#### 2.2.3 Treatment stabilization

Upon treatment stabilization, patients recover social conditions and cessation of illicit opiate use. When it is not possible to conduct physical examination on-site and assess behavior and mental state in person, indicators of treatment stabilization can be gauged through medical record, dosing records, and leaving applications. This study applied the total number of days attended during the past 30 days ( $\chi_{1}$ ), result of urine drug screen using a score ( $\chi_{2}$ ) to indicate social conditions, cessation of illicit opiate use, respectively. Therefore, treatment stabilization was defined as the sum of $\chi_{1}$ and $\chi_{2}$ .

#### 2.2.4 Likelihood of treatment discontinuation

The likelihood of treatment discontinuation was indicated if the patient did not want to increase the methadone dose, whereas the likelihood of treatment continuation was defined as the opposite. The likelihood of discontinuation was defined as the proportion of co-occurring reduced methadone doses and opiate-positive urinalysis results. The proportion was normalized against the total number of outpatient visits and denoted as $\chi_{3}$ .

#### 2.2.5 Physician-prescribed methadone dose

For a given patient, let to be the number of outpatient visits and $D_{t}$ be the methadone dose prescribed for visit t, serving as the observed methadone dose. The first dose of methadone prescribed at the outpatient visit after January 1, 2019, was defined as the baseline dose.

#### 2.2.6 Optimized methadone dose

The optimized methadone dose was determined by considering seven predictor variables, including HIV positivity, referral by the criminal justice system, 90-day urine opiate test results, last methadone dosage taken, willingness to receive treatment, 7-day treatment adherence, and 30-day treatment adherence. We built a model to calculate the optimal methadone dose using a two-layer feed-forward neural network (APP; MATLAB R2021a). For each patient, the optimal methadone dose specific to the individual was predicted using these seven predictor variables, and it was updated over time whenever data from the latest time unit was obtained. Each patient was referred to a block in the mathematical model. This enabled the model to address inter-individual variability, although it did not yet include variations in non-objective variables and pharmacokinetic among individuals.

#### 2.2.7 Co-morbid factors and demographic data

Patient characteristics, including sex, age, total treatment duration, HIV status, and referral by the criminal justice system were obtained from outpatient medical records. Patients referred by the criminal justice system were in mandatory opioid agonist treatment under deferred prosecution. Consequently, they had less autonomy to discontinue treatment, stricter requirements for the total number of days attended dosing during the past 30 days, and less autonomy to reduce methadone dose.

### 2.3 Statistical analyses

#### 2.3.1 Descriptive statistics

Data are represented as medians and interquartile ranges for continuous variables, such as total treatment duration, urine opiate test scores ( $\chi_{2}$ ), 30-day treatment adherence ( $\chi_{1}$ ), physician-prescribed methadone dose ( $D_{t}$ ), likelihood of treatment discontinuation ( $\chi_{3}$ ), and treatment stabilization ( $\chi_{1}+\chi_{2}$ ). Frequencies and percentages are used to represent categorical data such as sex, HIV status, and referral by the criminal justice system.

#### 2.3.2 Mixed modeling

Given that the 30-day treatment adherence ( $\chi_{1}$ ), 90-day urine opiate test scores ( $\chi_{2}$ ), and methadone dose varied at the individual level, random effects were modeled using generalized estimating equations. The fixed effects included in mixed modeling consisted of age, sex, total treatment duration, HIV status, referral by the criminal justice system, and likelihood of treatment discontinuation ( $\chi_{3}$ ). The physician-prescribed methadone dose ( $D_{t}$ ) served as the dependent variable. Generalized estimating equations were implemented to assess clustering of $\chi_{1}$ , $\chi_{2}$ and $D_{t}$ across patients using the SAS Enterprise Guide version 7.15 (SAS Institute Inc., Cary, NC, United States). Statistical significance was defined as a two-sided *p* < 0.05.

#### 2.3.3 Machine learning approach

Our study adopted neural network modeling, which is a nonlinear regression model (Supplementary Figure S1). The artificial neural network is a mathematical and computational model inspired by the nervous system of human brain. Complicated problems can be solved only using a nonlinear activation function. In this analysis, nonlinear activation function $2/\left(1+e^{-2x}\right)-1$ is default. After introducing the nonlinear activation function, the neural network can approximate any other complicated behavior.

The predicted variable was the physician-prescribed methadone dose. The simulation data used the same parameters as the mixed model, including person-days of dosage (*i* = 40,530), number of subjects (*j* = 71), age, sex, total treatment duration, HIV status, referral by the criminal justice system, 30-day treatment adherence ( $\chi_{1}$ ), urine opiate test scores ( $\chi_{2}$ ), and likelihood of treatment discontinuation ( $\chi_{3}$ ). Using a machine learning approach, we tested whether the difference between the methadone dose determined by physicians and that determined by the model was within the allowable range. To estimate the performance of the machine learning model, we applied cross-validation (20). First, we randomly divided the original dataset into n mutually exclusive partitions and then trained the model using n − 1 partitions, followed by testing the trained model on the remaining partition. This procedure is repeated using different training and testing data partitions (n-folder cross-validation). In our study, we used 10-folder cross-validation. To build a high-quality regression model, in addition to the correlation coefficient, we determined the normality of the distribution of the differences between the model-optimized and physician-prescribed methadone doses.

## 3 Results

### 3.1 Descriptive statistics

Of the initial 96 eligible patients with opioid use disorder, 14 were excluded due to the use of buprenorphine treatment, 9 were excluded due to short (<30 days) treatment duration, and 2 had missing data regarding urine drug screening. A total of 40,530 daily dosing records and 1,508 urine opiate results obtained from 71 patients with opioid use disorder were analyzed. Among the remaining 71 study subjects, 90.1% were male (*n* = 64), and the average age was 45.0 ± 7.1 (mean ± SD) years. Among them, 9.9% were HIV-positive (*n* = 7) and 26.8% (*n* = 19) were referred by the criminal justice system (Table 1).

**Table 1:** Descriptive statistics for the 40,530 daily dosing records of opioid agonist treatment.

	N	%
Number of patients	71	
Number of dosing days	40,530	
Number of urinalyses	1,508	
Male	64	(90.1%)
Average age (years)	45.7	±7.1
Treatment duration in months^1^	9.0	(1.0–56.5)
Referred by the criminal justice system	19	(26.8%)
HIV-positive	7	(9.9%)
90-day urine drug screen		
Opiate-negative	20	(28.2%)
Opiate-positive	10	(14.1%)
Add-on test with opiate-negative result^2^	41	(57.7%)
Methadone dose^1^	45.0 mg	(30.0–68.0 mg)
Likelihood of treatment discontinuation^3^	0.02	(0.00–0.03)
7-day treatment compliance^1^	7.0 days	(6.0–7.0 days)
30-day treatment compliance^1^	25.0 days	(14.0–29.0 days)
Treatment stabilization^1,4^	39.0	(35.0–48.0)

^1^Continuous data with normal distribution are presented as mean ± SD. Continuous data with a non-normal distribution are presented as median (IQR). Categorical data are presented as N(%). ^2^For patients who had opiate-positive urine drug screen results, an add-on urine drug screen was performed 1 week after. ^3^Likelihood of treatment discontinuation is the proportion of co-occurrence of opiate-positive urinalysis results and reduced methadone dosage among total outpatient visits. ^4^Treatment stabilization is defined as the sum of the 90-day urine opiate test score and 30-day treatment compliance.

**Figure 2:**
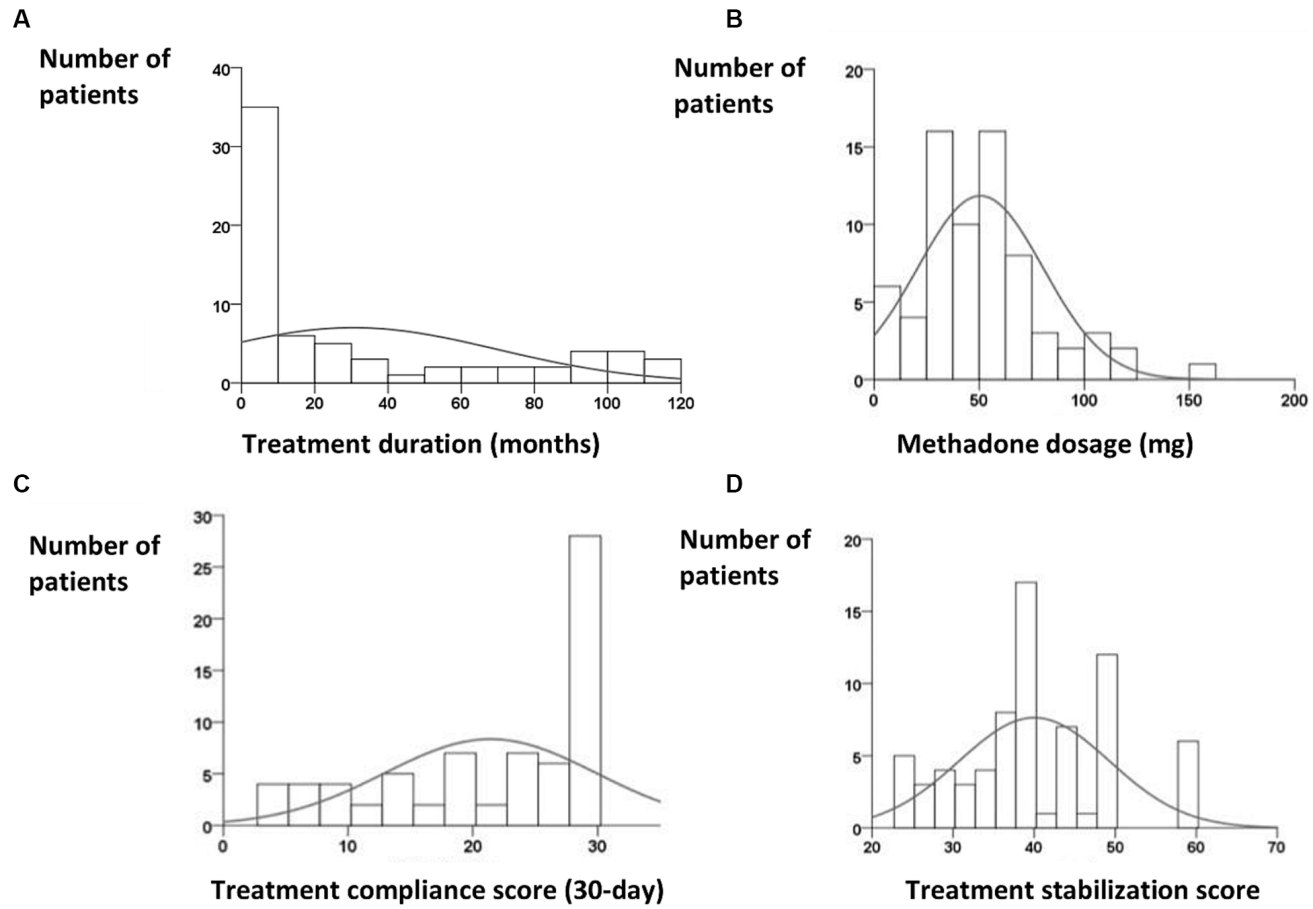
**(A)** Skewed distribution of treatment duration, with a peak occurring between 1 and 10 months [median, 9 months; interquartile range (IQR), 0–56.5 months]. **(B)** Distribution of methadone dosage revealed a peak between 30 and 60 mg (median, 45 mg; IQR, 30–68 mg). **(C)** Distribution of 30-day treatment compliance (median, 25 days; IQR, 14–29 days). **(D)** Distribution of treatment stabilization, which represents the sum of the 90-day urine opiate score and 30-day treatment compliance.

The median treatment period was 9 months [interquartile range (IQR), 0–56.5 months]. The scatter plot of treatment duration appeared U-shaped (Figure 2A). Most patients had treatment durations of <10 months or 90–120 months. The median methadone dosage prescribed by physicians was 45 mg (IQR, 30–68 mg) (Figure 2B), which was less than the 60-mg maintenance dose of methadone. The median 7-day treatment adherence was 7 days (IQR, 6–7 days), and the median 30-day treatment adherence was 25 days (IQR 14–29 days) (Figure 2C), which revealed fair treatment retention. We considered that 22.5% (*n* = 16) of the participants were unwilling to receive treatment. The median treatment stabilization score was 39 (IQR, 35–48) (Figure 2D), which was the sum of the 90-day urine opiate test score and 30-day treatment compliance.

### 3.2 Mixed modeling

The purpose of the linear mixed model was to estimate random effects and fixed effects for data with intra-individual variability and without intra-individual variability, respectively. Random effects were estimated for the variables with intra-individual variability, including 90-day urine opiate test scores, and methadone dose. Fixed effects were calculated for age, sex, total treatment duration, HIV status, referral by the criminal justice system, and likelihood of treatment discontinuation.

Patients with opiate-positive urinalysis results had lower doses of methadone than those with opiate-negative urinalysis results [*β* = 0.45, 95 % confidence interval (CI) 0.16–0.73]. Those without urine opiate test results in the last 90 days also had lower doses of methadone compared with those with opiate-negative urinalysis results (*β* = 0.27, 95 % CI 0.10–0.45) (Table 2). These findings suggest an inverse relationship between urine opiate test scores and methadone dose.

**Table 2:** Results of the mixed model using generalized estimating equations for methadone dosage.

	Methadone dosage				*p*-value
	β	SE	95%CI		
Urine drug screen within 90 days					
Opiate-negative	Reference 1				
Opiate-positive	0.45	0.15	0.17	0.74	*p* < 0.01
Missing urine drug screen	0.27	0.09	0.1	0.45	*p* < 0.01
Likelihood of treatment discontinuation^1^	3.67	6.71	−9.48	16.82	0.58
Treatment stabilization^2^	0.00	0.01	−0.02	0.03	0.96

Adjusted for age, sex, treatment duration, HIV positivity, and referral by the criminal justice system. ^1^Likelihood of treatment discontinuation is the proportion of co-occurrence of opiate-positive urinalysis results and reduced methadone dosage among total outpatient visits. ^2^Treatment stabilization is defined as sum of the 90-day urine opiate test score and 30-day treatment adherence.

To test the hypothesis that the likelihood of discontinuation delineates methadone doses, the methadone dosage was classified into three trends: decreasing, unchanged, and increasing. Table 2 presented the outcomes of urine opiate tests in relation to the quantity of methadone administered, while Table 3 detailed the findings of urine opiate tests regarding a decrease in the quantity of methadone dispensed. The likelihood of discontinuation was associated with decreasing dosages (*β* = 0.002, 95 % CI = 0.000–0.081) (Table 3), which indicated that the odds ratio of decreasing dosage was 0.002 times lower with every increment in the likelihood of discontinuation. In other words, with every increment of likelihood of discontinuation, the odds ratio of decreasing dosage was reduced by 99.8%.

**Table 3:** Results of the mixed model using generalized estimating equations for decreasing methadone dose.

	Number of outpatient visits (%)	Median (IQR)	Decreasing methadone dose			*P*-value
			OR	95% CI		
90-day urine drug test score						
Opiate-negative	258(17.1)		1.00			
Opiate-positive	950(63.0)		0.41	0. 06	2.92	0.38
Add-on test with opiate-negative result^1^	300(19.8)		0.67	0.25	1.82	0.44
Likelihood of treatment discontinuation^2^		0(0–0)	0.002	6.92E-05	0.08	*P* < 0.01
Treatment stabilization^3^		39(35–48)	0.90	0.82	0.99	*p* < 0.05

Adjusted for age, sex, treatment duration, HIV positivity, and referral by the criminal justice system. ^1^For patients who had opiate-positive urine drug screen results, an add-on urine drug screen was performed 1 week after. ^2^Likelihood of treatment discontinuation is the proportion of co-occurrence of opiate-positive urinalysis results and reduced methadone dosage among total outpatient visits. ^3^Treatment stabilization is defined as the sum of the 90-day urine opiate test score and 30-day treatment compliance.

### 3.3 Machine learning approach

Correlation analysis between physician-prescribed methadone dose and the optimized methadone dose showed a mean correlation coefficient of 0.995 ± 0.003 (*p* < 0.001), indicating the difference is within the allowable range.

**Figure 3:**
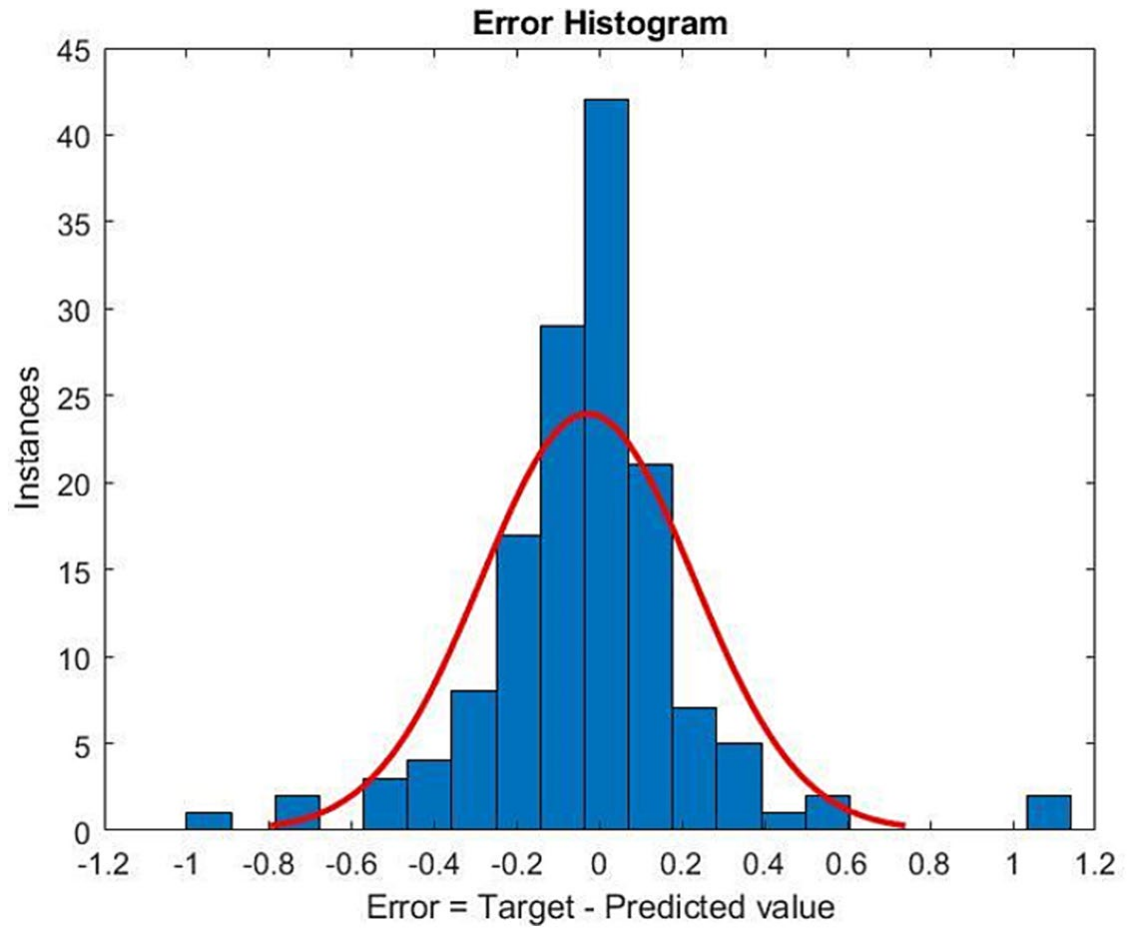
Histogram of the difference between the optimized methadone dose and the physician-prescribed methadone dose. The red curve represents the normal distribution fit. Target: optimized methadone dose. Predicted value: physician-prescribed methadone dose. Error: the difference between the optimized methadone dose and the physician-prescribed methadone dose.

The distribution of the difference between physician-prescribed and optimized doses showed normality at the center but did not show skewness (Figure 3).

## 4 Discussion

In this study, we investigated how methadone dosages are affected by urine opiate test scores, treatment adherence, and likelihood of treatment discontinuation. We further examined whether we could construct a model to predict dose adjustments for opioid agonist treatment.

Our study revealed that opiate-positivity and missing urine drug test results were associated with lower methadone doses. In our setting, patients were encouraged to increase methadone doses if the most recent urinalysis revealed opiate positivity. However, we found that opiate positivity and missing urine drug test results did not lead to an increase in methadone dose. This discrepancy could be related to patients’ wishes to reduce the methadone dose.

The findings reflected that individuals with lower methadone dosage and those who did not wish to increase the dosage as recommended by the treating physician, desired to use illicit opioids. Both the efficacy and likelihood of treatment discontinuation are considered in the decision to administer methadone. Our study demonstrates a relationship between the likelihood of discontinuation and an inadequate methadone dose. Previous studies addressed the patient-reported symptoms of abstinence syndrome, in which the patient’s claim of “enough” was not truly enough (22).

The strength of our study is the examination of the patients’ attitudes, particularly their likelihood of treatment discontinuation, which may predict an inadequate methadone dosage. We defined the likelihood of discontinuation by combining the coexisting conditions of abnormal urinalysis results and tapering methadone dosage. The advantage of our formula-based measurement is that it detects attitudes during treatment, which influences treatment retention (23).

Previous studies have implied that transportation, intake fees, closing time, and convenience of rapid intake are associated with treatment willingness (24). Future studies to establish automatic methadone dose adjustment should include quantifiable factors such as travel distance from accommodation to hospital, fees, operating hours, and convenience of parking and public transportation. Disinhibition of personality traits and negative emotionality predict premature termination (25). Therefore, it is helpful to conduct an initial personality assessment as a baseline indicator of treatment willingness. While willingness is crucial factor for treatment retention, it cannot serve as a predictor of methadone dosage. It is essential to incorporate withdrawal symptoms, cravings, objective and self-perceived adverse effect, co-existing physical and mental health conditions, the impact of co-administered medication (drug–drug interactions), genetic variations in metabolizing enzymes, hepatic and renal function into algorithmic models for predicting individual methadone dosage.

Our study adopted neural network modeling, a nonlinear regression model, and determined that a highly close relationship existed between predicted values and targets (correlation coefficient = 0.995 ± 0.003). Therefore, our model had a good predictive value.

This study has some limitations. First, the patients were screened for a urine opiates every 90 days, which was sparingly performed. A 90-day period is much longer than the opioid retention window. This means that heroin use might have been undetected depending on the substance used. In addition, urine opiate tests did not detect synthetic opioids. Second, participants were enrolled from a single institution. Future studies involving multiple institutes are needed to increase the sample size and improve the external validity. Third, our analysis did not include the signs and symptoms of withdrawal syndrome. Markers of the autonomic nervous system such as arterial blood pressure, heart rate, and respiratory rate should also be considered when determining opioid withdrawal levels (26, 27). Forth, the likelihood of discontinuation was predicted only based on urine opiate screening and whether the methadone dose was increased or not. Possible reasons for use of illicit opioids should be discussed with the patient together with assessing of the clinical symptoms, and likewise the reasons for not being willing to increase the dose. Then the likelihood of treatment discontinuation was related to also other factors than willingness to increase the dose. When on-site physical examination and in-person assessment of clinical symptoms was not feasible, obtaining the indicators of clinical symptoms was necessary to estimate the likelihood of discontinuation. Finally, patients who tested positive for opiate in urine drug screen within a 90-day period received lower methadone doses compared to those with opiate-negative results (β = 0.45 and 1, respectively). In contrast, individuals who did not provide urine specimens were associated with lower methadone doses, compared to those with opiate-positive results. Although there was a discernible relationship, the correlations were weak (β = 0.27 and 0.45, respectively). This might be due to Type I error.

In conclusion, we developed a model for methadone dose adjustment in patients with opioid use disorder by integrating urine opiate test results, treatment adherence data, and likelihood of treatment discontinuation. Further studies are needed to include other significant predictors such as clinical measurements, opioid withdrawal symptoms, adverse effects, co-morbid conditions, drug interactions and genetic heterogenicities in similar models to enhance the predictive value and accordingly clinical implications of such algorithm-based methods as a supplementary tool in clinical decision making on methadone dose adjustments.

## Data availability statement

The original contributions presented in the study are included in the article/Supplementary material, further inquiries can be directed to the corresponding author.

## Ethics statement

The studies involving humans were approved by the Ethics Review Committee of the Taichung Veterans General Hospital (project number *CF*-19714A). The studies were conducted in accordance with the local legislation and institutional requirements. The ethics committee/institutional review board waived the requirement of written informed consent for participation from the participants or the participants’ legal guardians/next of kin because The study adapted secondary database, in which the name and ID of an individual patient can not identified.

## Author contributions

PL: Validation, Writing – review & editing. T-YK: Formal analysis, Writing – original draft. I-CC: Writing – original draft, Conceptualization, Data curation, Formal analysis, Funding acquisition, Investigation, Methodology, Software. S-WL: Project administration, Writing – original draft. T-GC: Conceptualization, Writing – review & editing. H-LC: Resources, Writing – original draft. J-PC: Formal Analysis, Writing – review & editing.
