# Supplementary material for: Optimizing methadone dose adjustment in patients with opioid use disorder
Source: Front Psychiatry. 2024 Jan 8;14:1258029. doi: 10.3389/fpsyt.2023.1258029 (PMC10800821; doi:10.3389/fpsyt.2023.1258029)

Supplementary Figure S1

The architecture of 2-layer neural network model for optimizing methadone dose adjustment. Total seven predictor variables were considered as follows: HIV positivity, whether referral by criminal justice system,90-days urine opiate test scores, last methadone doses taken, likelihood of treatment discontinuation, 7-days treatment adherence and 30-days treatment adherence. The artificial neural network is a mathematical and computational model inspired by the nervous system of human brain. Complicated problems can be solved only using a nonlinear activation function. In this analysis, nonlinear activation function $2/(1+\mathrm{e}^{-2x})-1$ is default. After introducing the nonlinear activation function, the neural network can approximate any other complicated behavior.

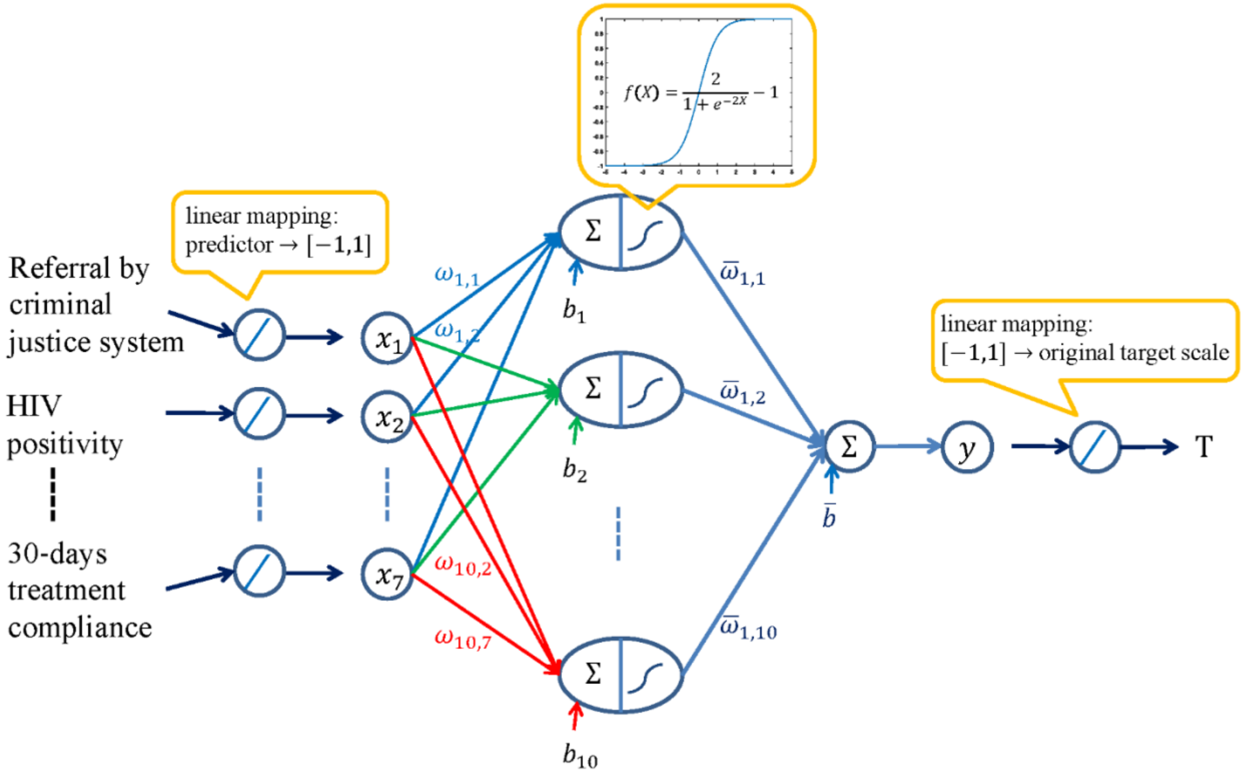

Supplement: Supplementary file 1 [file Presentation_1.PDF]
